# Antimicrobial Packaging from Potato Starch and Pectin with Citric Acid and Bioactive Compounds from Cashew Apple: Preparation, Characterization, and Application in Bread

**DOI:** 10.1021/acsomega.5c00409

**Published:** 2025-04-23

**Authors:** Janaína de Moura Fernandes, Cristiani Viegas Brandão Grisi, Rita de Cassia Andrade da Silva, Érica da Costa Monção, Géssica Alexandre de Barros, Sanierlly da Paz do Nascimento, Janeeyre Ferreira Maciel, Angela Maria Tribuzy de Magalhães Cordeiro, Neide Queiroz, Antônia Lucia Souza

**Affiliations:** a Programa de Pós-Graduação em Ciência e Tecnologia de Alimentos, Universidade Federal da Paraíba, João Pessoa, Paraiba 58050-085, Brazil; b Programa de Pós-Graduação em Química, Departamento de Química, Universidade Federal da Paraíba, João Pessoa, Paraiba 58050-085, Brazil; c Departamento de Engenharia de Alimentos, Universidade Federal da Paraíba, João Pessoa, Paraiba 58050-085, Brazil; d Departamento de Tecnologia de Alimentos, Universidade Federal da Paraíba, João Pessoa, Paraiba 58050-085, Brazil

## Abstract

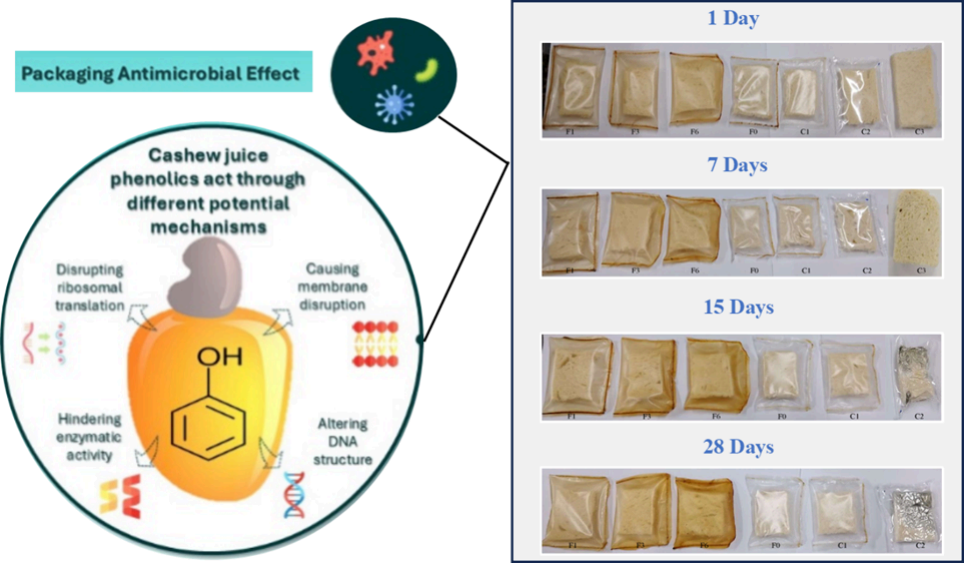

This study aimed to develop and characterize active antimicrobial films composed of potato starch and pectin, by incorporating inverted sugar as a plasticizer and bioactive compounds from cashew (CC) and citric acid (CA) as additives for application in bread packaging. Five treatments were formulated by the solution casting method: F0 (without CC-0.5% CA), F1 (1% CC-0.25% CA), F3 (3% CC-0.5% CA), F6 (6% CC-1% CA), and C1 (without CC and CA). Two other controls were used in the bread application (C2: low-density polyethylene and C3: unpackaged bread). Treatments with additives exhibited an increased water vapor permeability compared to C1; F6 showed the highest value (7.62 × 10^–4^ g H_2_O mm/m^2^ h mmHg). Conversely, C1 demonstrated superior tensile strength (21.13 MPa) compared to the other treatments, while films containing additives displayed heightened elongation (507.19%) relative to C1. Color parameters indicated a decrease in L* values (88.95), accompanied by an increase in a* (0.62) and b* (16.64) values for the high-concentration treatment (F6). Additionally, F6 degraded completely within 8 days. Therefore, the application of active films (F1 and F3) acted as antimicrobial packaging for bread, extending its microbiological stability 4-fold from 7 to 28 days. Future studies should explore the optimization of film formulations and their scalability for commercial applications.

## 1 Introduction

Active packaging is an innovative concept designed to meet both industry and consumer expectations. Its primary characteristic is its ability to interact with food or the surrounding environment by releasing or absorbing functional substances, such as antimicrobials, antioxidants, or moisture regulators.^1^ Researchers are also exploring biodegradable and renewable-source materials for developing polymeric packaging. Polysaccharides, proteins, and lipids, often edible, are the main materials studied for biopolymer production, used individually or in blends.^2^

Polysaccharide-based films, such as those made from starch and pectin, have been widely studied due to their abundance in nature, low cost, and ability to form films with suitable malleability and low permeability to oxygen and water vapor. Potato starch (*Solanum tuberosum* L.) is particularly promising for biodegradable packaging. It produces films with high oxygen and water vapor barrier properties, along with greater transparency, although it has a lower amylose content (20%) compared to wheat and corn starch (25 and 27%, respectively).^3^

Pectin, a biopolymer extracted from fruit residues (passion fruit, citrus peels, and apples), is widely used as a gelling agent in jams and jellies to prevent crystallization. Chemically, it is a heteropolysaccharide soluble in water and acid, primarily composed of d-galacturonic acid α-(1→4) linked to other sugars such as galactose, arabinose, and rhamnose.^4^ These polysaccharides can be used individually or in blends, offering a viable alternative to petroleum-derived polymers.^5^

In addition, natural additives are increasingly investigated for their potential applications in biodegradable packaging.^1,5^ The bioactive compounds from cashew and citric acid, due to their antimicrobial properties, represent promising natural additives. The cashew pseudo fruit (*Anacardium occidentale* L.) is rich in bioactive compounds, including vitamin C, sugars such as fructose, sucrose, and glucose, dietary fiber, organic acids (citric acid and lactic acid), and polyphenols such as tannins. These compounds have demonstrated antimicrobial activity against pathogens including *Listeria monocytogenes* and *Staphylococcus aureus* (*S. aureus*).^6−8^ Citric acid, a poly(carboxylic acid) found in citrus fruits such as lemons and oranges, also exhibits antimicrobial activity.^9^ It has been shown to inhibit *Enterococcus faecalis*, *Pseudomonas aeruginosa*, *Klebsiella pneumoniae*, and *Staphylococcus aureus*.^10^

The antimicrobial efficacy of these compounds depends on their concentration and may require further optimization for application in biodegradable packaging. Phenolic compounds such as tannins disrupt bacterial membranes and inhibit metabolic enzymes, showing antibacterial activity against *Escherichia coli*, at a minimum inhibitory concentration (MIC) of 512 μg/mL.^11^ Cashew apple juice shows an MIC value of 15.6 μg/mL against *S. aureus*, while citric acid showed antimicrobial activity at 0.06 g/mL, both when tested independently.^12,13^ These findings demonstrate the potential of cashew bioactive compounds and citric acid as natural antimicrobial agents, supporting their use in active packaging systems for food preservation.

In this context, bioactive compounds from cashews, especially phenolic compounds, and citric acid have been studied for their antimicrobial properties, making them promising natural additives for food preservation. This study hypothesized that these compounds could effectively control microbial growth in bread and that their synergistic antimicrobial effects may enhance the efficacy of active packaging. To the best of our knowledge, no previous studies have explored the development of antimicrobial films using potato starch, passion fruit pectin, inverted sugar, cashew bioactive compounds, and citric acid as the primary components. Therefore, this study aimed to develop and characterize active packaging based on potato starch and passion fruit pectin through incorporation of inverted sugar as a plasticizer and bioactive compounds from cashew and citric acid as additives. Additionally, the films were applied to bread and their effects were monitored for 28 days to evaluate microbial stability.

## 2 Materials and Methods

### 2.1 Materials

Cashew fruits, lemons, passion fruits, potato starch, ovalbumin, sucrose, citric acid, and loaves of bread were purchased from a local market in João Pessoa, PB, Brazil. Citric acid was used without further purification.

#### 2.1.1 Obtaining Inverted Sugar

To obtain inverted sugar, 100 mL of distilled water, 100 g of sucrose, and 3.6 mL of Tahiti lemon juice were weighed. The sucrose and water mixture were manually homogenized and heated to 120 °C for complete dissolution. Lemon juice was then added, and the solution was boiled for 5 min. The resulting inverted sugar was cooled in a glass container and stored at room temperature.^14^ It exhibited 5.43 g/L reducing sugars, 78.30° Brix, and a pH of 5.50.

#### 2.1.2 Pectin Extraction from Passion Fruit Peel

Yellow passion fruits were washed under running water and sanitized before halved to remove seeds and pulp. The entire peel, including the inner layer (albedo) and the colored outer layer (flavedo), was cut into smaller pieces. A total of 200 g of chopped peel, 500 g of distilled water, and 20 g of lemon juice (used as an acidifier) were weighed. The peel was first heated in water until it softened. Lemon juice was then added, and the mixture was heated again for an additional 10 min. The resulting pectin extract was transferred to a glass container, cooled to room temperature (25 °C), and stored at 4 °C.^15^

#### 2.1.3 Obtaining Bioactive Compounds from Cashew

Cashew fruits were washed under running water, submerged in a chlorine solution (100 mg/L free chlorine), rinsed, and then frozen at −18 °C. After 12 h of thawing at 5 °C, the cashew pulp was obtained by manual maceration. Foam production followed the method described by Dehghannya and collaborators^16^ with adaptations in the concentration of ovalbumin and foam thickness. To extract bioactive compounds, the cashew pulp was mixed with 2% (w/w) egg albumin and aerated in a domestic mixer at the maximum speed for 10 min. The resulting foam was spread onto aluminum plates (15 × 1.5 cm) in a uniform layer of 1.5 cm thickness. Drying was performed in an air circulation oven at 60 °C for 4 h until a constant weight was achieved.^17^ The dehydrated material was removed from the plates using a spatula, macerated, and stored in polyethylene containers wrapped in aluminum foil. The samples were then refrigerated at 4 °C until further use.

### 2.2 Film Production

The films were produced using the casting method described by Araújo et al.^15^ Potato starch (4%, m/m) was dissolved in distilled water, along with passion fruit pectin extract (1%, m/m), inverted sugar (2%, m/m), citric acid (0.25, 0.5, and 1%, m/m), and bioactive compounds from cashew (1, 3, and 6%, m/m). The prepared treatments were F0 (without cashew bioactive compounds (CC) and 0.5% citric acid (CA)), F1 (1% CC and 0.25% CA), F3 (3% CC and 0.5% CA), F6 (6% CC and 1% CA), and C1 (without CC and CA).

The mixture was homogenized for 60 min using a magnetic stirrer at room temperature (25 °C). It was then heated to 70 °C under constant stirring until starch gelatinization was achieved. A total of 40 g of each solution was poured into acrylic Petri dishes (150 × 15 mm). The plates were then placed in an air-circulating oven at 40 °C for 14 to 16 h. The obtained films were conditioned at 25 °C and 75% relative humidity (RH) for 2 days before being removed from the acrylic plates and evaluated.

### 2.3 Film Characterization

#### 2.3.1 Thickness and Mechanical Properties

Film thickness was measured using a portable micrometer with an accuracy of 0.001 mm. Ten random measurements were taken 60 mm from the film edges at room temperature. The average thickness was used to evaluate the mechanical properties, and results were expressed in millimeters (mm).

Tensile strength (MPa) and elongation at break (%) were determined using a SHIMADZU static testing instrument (Brazil), following the standard method D882-12.^18^ Rectangular specimens (100 mm × 15 mm) were tested with an initial grip separation of 50 mm and a crosshead speed of 12.5 mm/min. Thickness for each specimen was measured at five different points using a digital caliper, and 10 replicates were analyzed for each treatment.

#### 2.3.2 Water Vapor Permeability (WVP)

The samples were characterized using the gravimetric method according to ASTM E96/E96M-16.^19^ Films, 5 cm in diameter, were used to seal the open end of acrylic containers filled with silica gel. These containers were placed in a desiccator at 25 °C and 75% RH (saturated NaCl solution). Sample weights were recorded every 24 h over a period of 7 days. Water vapor permeability was calculated by eq 1, and results are expressed in gH_2_O mm/m^2^ h mmHg.
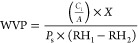
1where *C_i_* is the slope of the line representing the weight gain of the system over time; *A* is the film area (m^2^); *X* is the film thickness (mm); *P*_s_ is the saturation pressure of water vapor at 25 °C (22 mmHg); RH_1_ is the relative humidity in the chamber (75%) and RH_2_ is the relative humidity inside the acrylic container (0%).

#### 2.3.3 Solubility

Water solubility was determined following the methodology described by Nordin et al.^20^ The films were cut into 2 × 2 cm squares, and the initial dry matter content was measured by drying the samples in an air circulation oven at 105 °C for 24 h. After the first weighing, the samples were immersed in 50 mL of distilled water in a 250 mL Erlenmeyer flask and agitated at 100 rpm (MultiShaker) for 24 h at 25 °C. The undissolved matter was then dried at 105 °C for 24 h, and the final mass was recorded. The solubility, expressed as a percentage, was calculated using eq 2.
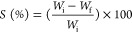
2where *W*_i_ is the initial mass of the dry material and *W*_f_ is the final mass of the undissolved dry material.

#### 2.3.4 Instrumental Color

The color of the films was measured using a GRETAG MACBETH-COLOR-EYE 2180 colorimeter with a standard illuminant D65 and a 10° observer. The films were placed on a standard white plate, and color measurements were recorded on a CIELAB scale. L* represents luminosity (0 = black, 100 = white), while a* and b* correspond to chromaticity coordinates, with a* ranging from green (−) to red (+) and b* ranging from blue (−) to yellow (+).^21^ For each film, the average of five random measurements with three replicates was used. The color difference (Δ*E*) was calculated relative to the control (C1) (*L*_standard_* = 95.01; *a*_standard_* = −0.36; *b*_standard_* = 2.68) using eq 3.
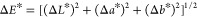
3where ΔL* = *L*_standard_ – *L*_sample_, Δa* = *a*_standard_ – *a*_sample_, and Δb* = *b*_standard_ – *b*_sample._

#### 2.3.5 Moisture Content and Water Activity

The moisture content was determined according to the methodology described by Adzaly et al.^22^ The films (2 cm × 2 cm) were weighed and placed in an oven at 105 °C for 24 h. The water activity of the films (4 cm × 4 cm) was measured directly using Aqualab Tev equipment.

#### 2.3.6 Degradability

The degradability of the films was assessed by measuring mass loss after exposure to natural soil microbiota over 15 days, with relative humidity between 65 and 75% and 25 °C. The samples were cut into rectangular shapes (2 cm × 2 cm), weighed, and buried in plastic containers at a depth of 15 cm. At 4, 8, and 15 days, the films were retrieved using tweezers, washed with distilled water to remove soil residues, and dried in an oven at 40 °C for 24 h.^23^

The percentage degradation was calculated using the following equation:
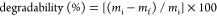
4where *m*_i_ is the initial film mass (g) and *m*_f_ is the final dry film mass (g).

#### 2.3.7 Thermogravimetric Analysis (TGA)

Thermogravimetric analysis of the films was conducted using a SHIMADZU DTG-60H thermal analyzer. A nonisothermal test was performed on 5–7 mg of the sample, which was placed in an alumina crucible and heated in a synthetic air atmosphere (50 mL/min) at a heating rate of 10 °C/min from 25 to 800 °C. Thermograms representing weight loss as a function of temperature were generated and analyzed using OriginPro software (version 8.5, OriginLab Corporation, Northampton, MA, USA).

#### 2.3.8 FTIR Analysis

The analyses were performed using a Fourier transform infrared spectrophotometer (FTIR) (SHIMADZU model IR Prestige-2, Japan) with the attenuated reflectance (ATR) method. Spectra were recorded in the range of 4000–600 cm^–1^ with a resolution of 4 cm^–1^ and 40 scans, according to ASTM D5477-11.^24^ Autobaseline correction was applied to all spectra before curve fitting. Fourier self-deconvolution spectra were generated using IR Solutions software (Shimadzu), and peak fitting was performed with OriginPro software (version 8.5, OriginLab Corporation, Northampton, MA).^25^

#### 2.3.9 Efficacy of Active Films in Loaves of Bread

The four active film formulations, F0 (without CC and 0.5% CA), F1 (1% CC and 0.25% CA), F3 (3% CC and 0.5% CA), and F6 (6% CC and 1% CA), along with three controls, C1 (without CC and CA), (C2: low-density polyethylene), and C3 (unpackaged bread), were subjected to an *in vivo* evaluation of antimicrobial efficacy in bread packaging. The loaves were heat-sealed in packaging (3 cm × 5 cm) and stored at 25 °C (75% RH). Samples were visually inspected for color change and fungal growth for periods of 0, 7, 15, and 28 days.^20^ After 28 days, the bread samples were analyzed for microbial contamination based on RDC 724 of 2022, assessing the presence of *Salmonella* spp. (absence in 25 g) and molds and yeasts (≤10^4^ CFU/g).^26^

##### 2.3.9.1 Sample Preparation

After 28 days of storage, 10 g of each sample was homogenized in 90 mL of 0.1% peptone water for 1 min in Stomacher bags. The analyses were performed in triplicate. Serial dilutions were then performed for microbial analysis, except for *Salmonella* spp., for which homogenization was carried out using lactose broth.^27^

##### 2.3.9.2 Total and Thermotolerant Coliforms

To determine the most probable number of total and thermotolerant coliforms, a presumptive test was performed. A total of 1 mL of each dilution was inoculated into three tubes per dilution. Each tube contained 10 mL of lauryl sulfate tryptose (LST) broth (HIMEDIA) and Bright Green lactose broth, along with an inverted Durham tube. The tubes were incubated at 35–37 °C for 48 h. As no contamination was detected, the analysis was terminated.^28^

##### 2.3.9.3 *Salmonella* spp.

To detect *Salmonella* spp*.*, 10 g of each sample was pre-enriched in a sterile Erlenmeyer flask containing 90 mL of lactose broth and incubated at 35 °C for 24 h. After incubation, selective enrichment was performed, transferring 0.1–10 mL of Rappaport–Vassiliadis broth and 1–10 mL of selenite cystine broth, followed by incubation at 35 °C for 24 h in an oven. Subsequently, differential plating was performed by streaking a loopful from each broth onto bismuth sulfite agar (HIMEDIA), Salmonella Shigella agar (KASVI), and xylose-lysine-deoxycholate agar (HIMEDIA).^29^ The plates were incubated upside down at 35–37 °C for 24 h.

##### 2.3.9.4 Mesophilic Aerobic Microorganisms

The presence of mesophilic aerobic microorganisms was evaluated using the pour-plate technique. A total of 1 mL of each dilution was transferred in a Petri dish, followed by the addition of 10 mL of standard count agar–PCA (Difco). The mixture was homogenized, and after solidification of the agar, the plates were incubated upside down at 35 °C for 48 h in an oven.^30^ Colony forming units (CFU) were counted using a colony counter, and the results were expressed in CFU per gram (CFU/g).

##### 2.3.9.5 Molds and Yeasts

For the determination of molds and yeasts, 10 g of each sample was quantified in 90 mL of sterile 0.1% peptone water under aseptic conditions. After homogenization, aliquots (0.1 mL) were placed on the dry surface of potato dextrose agar (HIMEDIA), acidified with 10% tartaric acid (VETEC) to pH 3.5.^23^ The inoculum was spread, and the plates were incubated right side up at 25 °C for 5 days. The reading was performed, and the results were expressed in CFU per gram (CFU/g).

### 2.4 Statistical Analysis

Results were expressed as means ± standard deviation (*n* = 3). Data were subjected to one-way analysis of variance (ANOVA), and Tukey’s test was applied to determine significant differences at a 95% confidence level (*p* < 0.05), using Assistat software version 7.7. Graphics were generated using OriginPro software version 8.5 (OriginLab Corporation, Northampton, MA, USA).

## 3 Results and Discussion

### 3.1 Physical and Mechanical Characterization of Films

Film thickness ranged from 0.08 to 0.25 mm, with the highest value observed in formulation F6 and the lowest in the control without additives C1. This variation may be attributed to the addition of bioactive compounds. A previous study has shown that the incorporation of various substances can increase film thickness, which in turn affects their functional properties.^31^

In this study, increasing the concentration of natural additives also led to a rise in the total solid content of the films, which ranged from 75.45 to 86.83 g/100 g. The film F0 containing only citric acid (CA) exhibited a moderate increase in thickness (0.11 mm); however, this increase was smaller compared to formulations containing cashew compounds (CC). As a result, films with CC exhibited greater thickness than the control (C1).

The thickness of the films was influenced by factors such as the composition, the viscosity of the film-forming solution, and the volume of solution spread on the drying plate.^32^

Tensile strength (TS) values ranged from 1.21 to 21.13 MPa. The highest TS was recorded for the control film (C1), which did not contain natural additives. When compared to the control, the addition of natural additives led to a reduction in tensile strength, with formulation F6 showing a decrease of up to 94.27. Similar results to F6 were reported by Istiani et al.^33^ in films based on starch, glycerol, and citric acid.

The reduction in tensile strength with increasing concentrations of natural additives may be attributed to the presence of bioactive compounds and ovalbumin in the CC. The hydroxyl groups in these materials may disrupt hydrogen bonds within the film matrix.^34^ As a result, the additives reduced the direct interactions and packing between the starch chains, thereby increasing the flexibility of the films.

This behavior was previously reported by Jawad et al.,^35^ who observed similar effects in polymeric films containing bioactive compounds. To mitigate the reduction in mechanical strength, the authors proposed incorporating chitin nanofibers (CNF) as a reinforcing agent in future formulations, with the aim of improving both elongation and tensile strength of the polymeric matrix. Recent studies have confirmed the effectiveness of CNF in enhancing the structural properties of films. Wang et al.^36^ reported a significant increase in tensile strength with CNF addition, with the most notable improvement observed at a 30% concentration. Furthermore, elongation at break increased by up to 35% with the incorporation of 10% CNF.

In addition to chitin, eucalyptus nanofibers have also been studied as a structural reinforcement in biodegradable films. De Oliveira et al.^37^ developed thermoplastic films reinforced with eucalyptus nanofibers and observed considerable improvements in tensile strength. The films also showed reduced water solubility, enhanced biodegradability, and preserved texture and moisture in food products such as cookies. Therefore, the incorporation of chitin or eucalyptus nanofibers represents a promising strategy to optimize the film matrix by providing structural reinforcement without compromising antimicrobial activity.

The elongation at break (ε) of the films ranged from 9.73 to 59.08%. The highest values for this parameter were observed in films containing natural additives. When comparing these formulations to the control film without additives, an increase of up to 507.19% was observed in the ε parameter for F3. This suggests that the additives exerted a plasticizing effect, enhancing the mobility of polymer chains, which in turn weakened the matrix and improved its flexibility. Similar behavior was reported by Huang et al.,^38^ who found that increasing the concentration of ovalbumin in packages made with ovalbumin and k-carrageenan led to a higher degree of matrix disorder. This structural change contributed to increased elongation at break and decreased tensile strength.

Films must function as protective barriers against small molecules such as water vapor, which can alter food composition and lead to undesirable effects.^39^ The WVP values of the films ranged from 0.26 to 7.62 × 10^–4^ gH_2_O mm/m^2^ h mmHg. Formulations containing higher concentrations of natural additives were more permeable to water vapor when compared to the control film without additives (C1). Basiak et al.,^40^ when producing films with potato starch and no additives, reported low permeability to water vapor. This was attributed to reduced wettability and lower water vapor absorption, which in turn was associated with the lower amylose content in potato starch compared to manioc starch.

The water solubility of the films ranged from 25.87 to 34.32%. Overall, the films remained visually intact after 24 h of homogenization. Solubility values decreased as the concentration of CC increased, likely due to the presence of ovalbumin, which has hydrophobic characteristics. This property makes the films suitable for packaging applications. Similar results were reported by Lima et al.,^41^ who observed reduced solubility in cornstarch and chitosan films with varying concentrations of cashew shell extract. Yazicioglu^42^ also noted a decrease in solubility when increasing CA concentrations in starch-based films. Consequently, films with low water solubility can be indicated for packaging foods with a high moisture content.

### 3.2 Instrumental Color

Color parameters influence consumer expectations regarding the visual appearance and overall acceptance of a product. As visual color perception is highly subjective, it can be influenced by environmental lighting conditions or the materials used.^43^ The developed films exhibited a homogeneous appearance, were free of insoluble particles, and showed no signs of ruptures. Consequently, they showed excellent malleability and could be easily removed from the plates after drying. The color measurements for all film samples are presented Table 1.

**Table 1:**
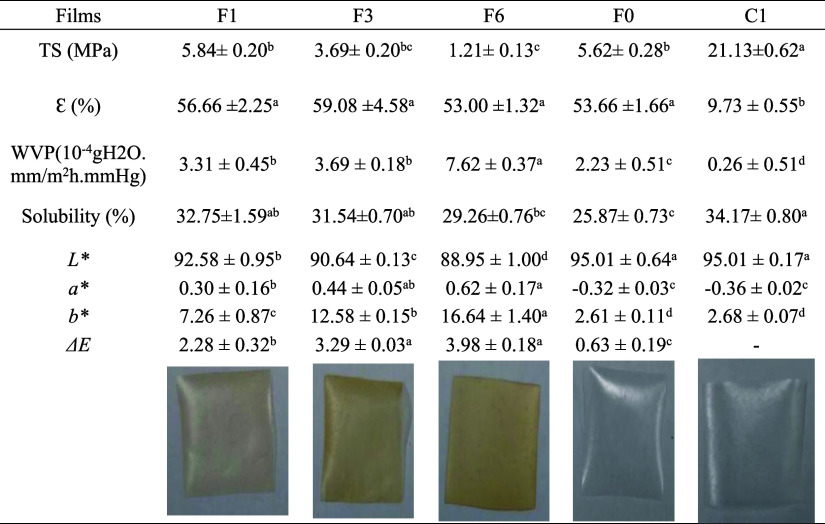
Structural Properties of Films: Tensile Strength (TS), Elongation at Break (ε), Water Vapor Permeability (WVP), Solubility, and Colorimetrya

a Values are expressed as means (*n* = 3) ± standard deviation. Means followed by the same letter on the same line are not significantly different from each other based on the Tukey test at a 5% significance level (*p* < 0.05). Formulations: F0 (without CC-0.5% CA), F1 (1% CC-0.25% CA), F3 (3% CC-0.5% CA), F6 (6% CC-1% CA), and C1 (without CC and CA).

The L* values of the films ranged from 88.95 to 95.01. These results indicate that film luminosity (L*) significantly decreased as the concentration of natural additives increased. Additionally, the total color difference (Δ*E*) increased. Despite this, the transparency of the films was visually maintained. The observed changes in the total color difference and brightness between formulations are likely due to the higher concentrations of natural additives, which may reduce light incidence.^44^ Folentarska et al.,^45^ in their development of ternary films composed of potato starch, lipids, and proteins, reported L* values similar to those found in the present study. Moreover, the reduction in L* suggests that the light-blocking characteristics of CC and CA could enhance food preservation, particularly in products sensitive to light exposure.

The a* coordinate showed very low and negative values, indicating a tendency toward a grayish hue, especially in C1 and F0 films, which did not contain CC and thus lacked pigments. This negative value can be attributed to the transparency and high luminosity of these formulations. The transparency of the films is noticeable to the naked eyes, a key feature for packaging that aims to showcase the food. In the b* coordinate, a significant increase in values was observed as the CC concentration increased, indicating a shift toward a yellowish hue, characteristic of the cashew pulp. This yellow tone could be advantageous for packaging products where warm colors (yellow/orange) enhance the perception of naturalness and ripeness, such as with fruit-based products.^46^

The most significant differences in Δ*E* color were seen in films with CC. Formulations with higher concentrations of CC and CA showed a marked increase in the color parameters, with the F6 formulation exhibiting a notable color change (Δ*E* = 3.98). However, despite these color changes, the films with CC retained their transparency. This suggests that natural colorants can modify the film’s aesthetics without compromising its transparency, an important trait in food packaging. These findings align with those of Alqahtani et al.,^47^ who also observed that increasing the concentration of date seed powder in films led to lower L* values and higher a* and b* values. In essence, films with higher concentrations of natural additives became darker and more intensely yellow, likely due to carotenoids in CC, still maintaining transparency compared to C1.

### 3.3 FTIR Analysis

**Figure 1:**
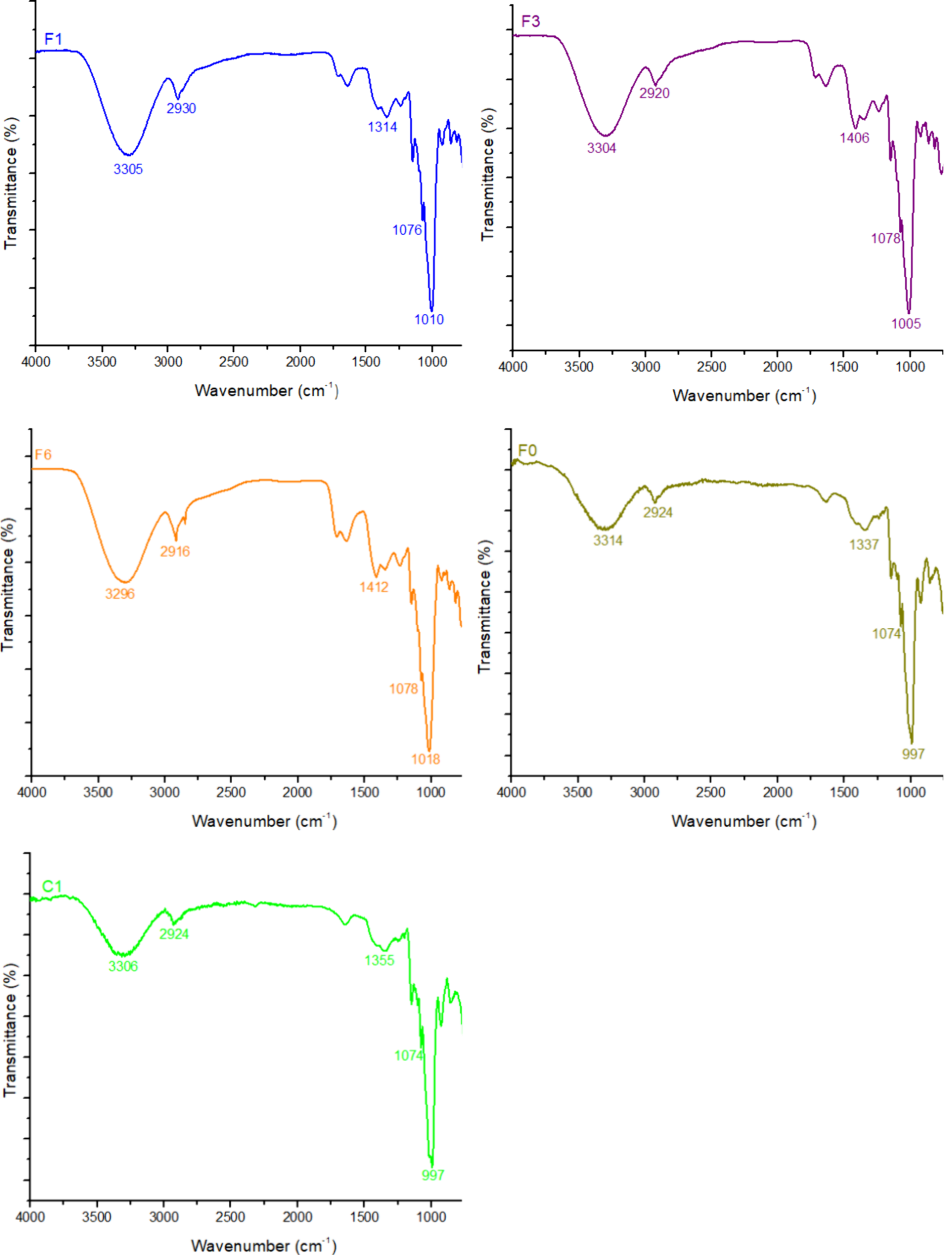
Medium infrared spectra of the films F0 (without CC-0.5% CA), F1 (1% CC-0.25% CA), F3 (3% CC-0.5% CA), F6 (6% CC-1% CA), and C1 (without CC and CA).

The FTIR technique was employed to identify and analyze the functional groups present in the films. The spectra, shown in Figure 1, exhibited characteristic bands in the region from 3296 to 3314 cm^–1^, corresponding to the stretching vibration of the O–H group.^48^ Bands observed between 2916 and 2930 cm^–1^ were attributed to the stretching of the C–H bond.^49,50^ The additives incorporated into the formulations, which contain phenols and flavonoids, possess hydrophilic functional groups, such as hydroxyl, ether, and carbonyls.^51^ The O–H, C–O, and C–O–C bonds in both the additives and the films contribute to intermolecular interactions, forming hydrogen bonds that significantly influence the mechanical properties of films.^52^

Bands in the region from 1337 to 1412 cm^–1^ are likely associated with C–H angular deformation of the polysaccharides.^53^ Additionally, the bands in the region 1074–1078 cm^–1^ are characteristic of the stretching of the C–O–C group, which is related to the glycosidic bonds found in polysaccharides, such as potato starch, as well as to ring bonds.^54^

The bands in the region of 997–1018 cm^–1^ are attributed to the angular deformation of the C–O group, a characteristic of carbohydrates such as fructose and glucose,^55^ which are present in the composition of the films. This region serves as a fingerprint for distinguishing molecules,^56^ making the differentiation clearly evident in the obtained spectra.

### 3.4 Thermal Analysis (TG/DTG)

**Figure 2:**
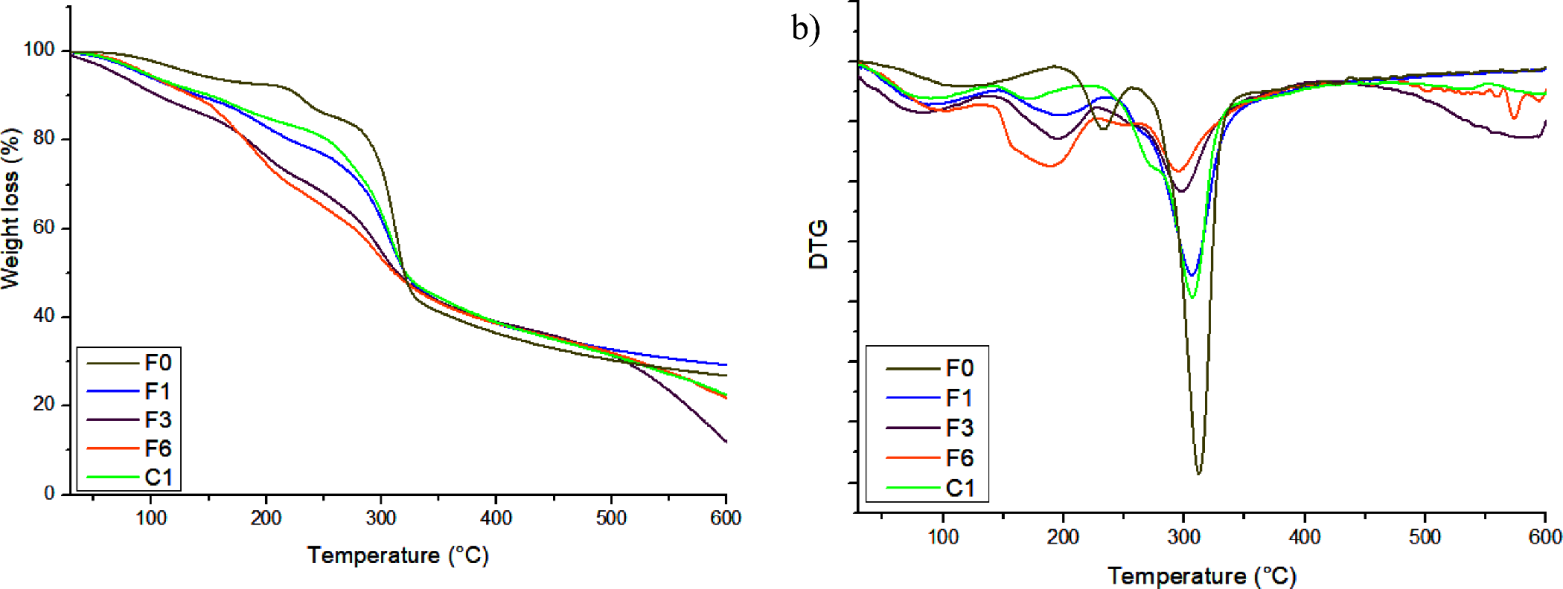
TG (a) and DTG (b) curves of the films. Formulations: F0 (without CC-0.5% CA), F1 (1% CC-0.25% CA), F3 (3% CC-0.5% CA), F6 (6% CC-1% CA), and C1 (without CC and CA).

The thermal behavior of the films was analyzed through TG/DTG thermogravimetric analysis, with results presented in Figure 2. The thermogravimetric curves (TG/DTG) of the films exhibited 3–4 stages of thermal degradation. In all the formulations, a similar pattern was observed during the first stage of mass loss (45.08–171.36 °C), which is attributed to the evaporation of water from films.^48^ The highest mass loss was recorded for the F6 film (16.46%), while the F1, F3, F0, and C1 films exhibited losses of 4.73, 8.77, 4.25 and 2.26%, respectively, in this first stage. Similar findings were reported by Assis et al.,^57^ who developed films with lycopene extract and lycopene nanocapsules, where the first mass loss event occurred at 110 and 150 °C. These results suggest that the produced films have favorable characteristics for food application, as they exhibit thermal stability at room temperature.

The second stage corresponds to the initial degradation of polysaccharides and ovalbumin (OVA), occurring from 104.62 to 360.91 °C in all films, as reported by Assis et al.^57^ In the F6 film, the peak observed at 360.91 °C corresponds to the highest mass loss (26.36%) while the F1, F3, F0, and C1 films showed losses of 4.64, 7.71, 3.67, and 1.97%, respectively, during this second stage. This greater mass loss confirms the reduced structural stability in the film, which is linked to higher concentration of additives in the formulation.

The third stage corresponds to thermal degradation from 213.17 to 501.41 °C. In the F0 film, the peak observed at 336.34 °C corresponds to the highest mass loss (29.77%) while the F1, F3, F6, and C1 films showed losses of 27.26, 6.50, 3.70, and 6.72%, respectively, during this stage. This phase is associated with chemical decomposition of the film components.^58^

The fourth stage corresponds to the degradation of the materials used in the films, occurring between 277.14 and 433.32 °C. In the F1 film, the peak observed at 330.98 °C corresponds to the highest mass loss (24.59%) while the F3 and C1 films showed mass losses of 2.44 and 11.93%, respectively, during this stage.

The total mass losses observed during thermal analysis for the F1, F3, F6, F0, and C1 films were 70.73, 88.43, 78.25, 82.71, and 73.17%, respectively. Films containing higher concentrations of natural additives exhibited greater weight loss as the temperature increased. Moreno et al.,^59^ when developing potato starch films with lyophilized bovine lactoferrin, reported that the presence and type of protein added significantly influenced film degradation. These effects were attributed to varying degrees of hydrogen bonding between starch hydroxyl groups and amino groups of the proteins. In the present study, the F1 formulation exhibited the lowest overall mass loss (70.73%), indicating better thermal stability. In contrast, formulations with higher additive concentrations showed reduced thermal stability.^60^

Therefore, this study demonstrates that natural additives significantly influence the thermal degradation behavior of biopolymeric films. Formulations with higher additive concentrations (F6 and F3) exhibited greater mass loss at elevated temperatures, suggesting reduced thermal stability likely due to structural modifications within the polymer matrix. In contrast, the F1 showed lower degradation, indicating better thermal resistance. These findings suggest that thermal stability of the films is closely associated with intermolecular interactions between starch, proteins, and the added compounds. This insight is particularly relevant for the design of biodegradable packaging materials, where optimizing thermal performance is essential for practical food applications.^59^

### 3.5 Degradability

Degradability tests were carried out to assess the degradation of the films over time following soil burial. Films with higher concentrations of natural additives (F6) showed complete degradation, after 8 days, representing the greatest loss among the tested formulations. In contrast, the control film (C1) showed a mass loss of 59.71% of its initial mass after 15 days (Table 2). Overall, the films with natural additives showed visible color changes after 4 days until the end of the analysis and fully degraded after 15 days. These results suggest that the presence of water in the soil facilitated penetration into the film matrix, causing swelling and promoting microbial activity, which in turn accelerated degradation. Conversely, the control film absorbed less water, which may have contributed to its slower degradation rate.

**Table 2:** Mass Loss (%) of Films during 15 Days of Soil Burial^a^

	mass loss (%)		
films	4 days	8 days	15 days
F1	46.37 ± 9.59^a^	78.02 ± 16.95^a^	100.00 ± 0.00^a^
F3	57.20 ± 8.54^a^	92.96 ± 7.15^a^	100.00 ± 0.00^a^
F6	61.71 ± 3.84^a^	100.00 ± 0.00^a^	100.00 ± 0.00^a^
F0	40.81 ± 22.68^a^	56.66 ± 32.26^ab^	83.26 ± 14.16^ab^
C1	44.22 ± 45.73^a^	24.18 ± 16.70^b^	59.71 ± 21.58^b^

a Values are expressed as means (*n* = 3) ± standard deviation. Means followed by the same letter on the same line are not statistically different from each other according to the Tukey test at a 5% significance level (*p* < 0.05). Formulations: F0 (without CC-0.5% CA), F1 (1% CC-0.25% CA), F3 (3% CC-0.5% CA), F6 (6% CC-1% CA), and C1 (without CC and CA).

Wang et al.^51^ verified that films made with starch, glycerol, and citric acid underwent rapid degradation within the first 15 days, although complete degradation occurred only after 30 days. The findings of the present study indicate that the incorporation of CC and CA enhances the biodegradability of the films. In general, higher concentrations of these natural additives resulted in greater biodegradation. This outcome indicates a promising approach to reducing the environmental impact associated with conventional plastic packaging produced from petroleum-based materials.^53^

The control of moisture and water activity in films is an important factor during the production of edible packaging, as these parameters influence both the shelf life of the material and the packaged food.^59^ Additionally, they can be related to the biodegradability of the films. The water activity values ranged from 0.61 to 0.67, while the moisture content varied between 13.17 to 24.55%. This variation may be attributed to the different proportions of CC and CA incorporated into each formulation. An increase in citric acid and CC concentration likely contributed to higher moisture levels due to the hygroscopic nature of natural additives. Based on these findings, it can be stated that the moisture content may have played a role in enhancing the biodegradability of the films, as those with higher moisture content exhibited greater mass loss during the observed period. In contrast, the water activity of the films did not appear to have a direct effect on biodegradability.

### 3.6 Antimicrobial Active Packaging

#### 3.6.1 Efficacy of Active Films in Loaves of Bread

**Figure 3:**
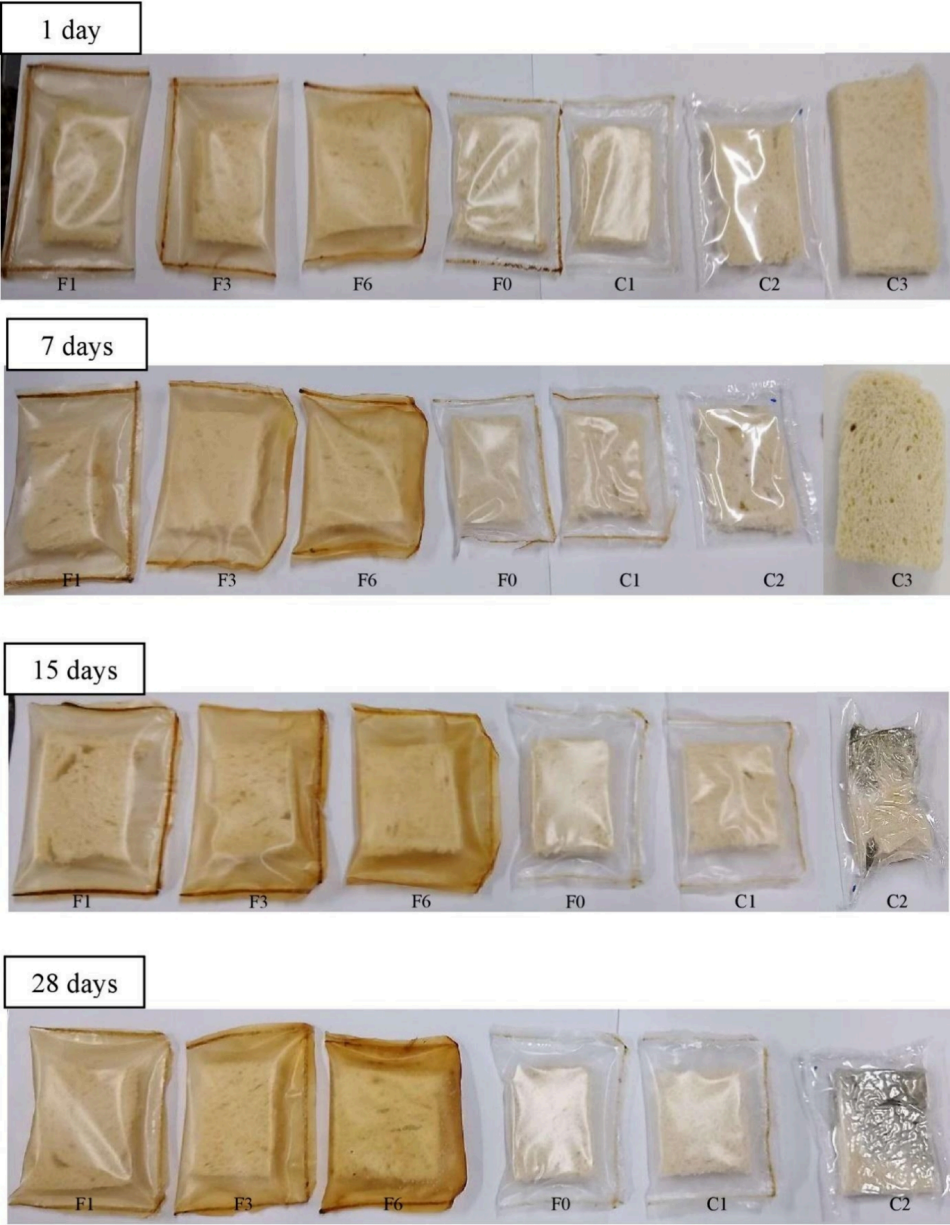
Bread packed: front area. Formulations: F0 (without CC-0.5% CA), F1 (1% CC-0.25% CA), F3 (3% CC-0.5% CA), F6 (6% CC-1% CA), C1 (without CC and CA), C2 (low-density polyethylene), and C3 (unpackaged breads, removed after 7 days of storage).

The microbial stability of bread packed with active films was evaluated over a 28-day period at 25 °C and 75% RH and can be seen in Figure 3. A visual inspection of both the packaging and the packaged product was conducted until the emergence of visible microorganisms.

The first signs of fungal growth on bread packed in low-density plastic were observed after 7 days, corresponding to the shelf life indicated on the commercial packaging. For the unpackaged loaves of bread (C3), significant fungal growth and dehydration were evident after 7 days, and microbiological analyses were not conducted. These results demonstrate that the active films extended the microbial stability of the bread compared to the low-density polyethylene and the unpackaged bread (C3).

In the case of the F6 formulation, a color change in the film was observed after 28 days of storage; however, this alteration did not affect the bread’s protection. After 28 days (four times the commercial shelf lives of the loaves), microbiological analyses were performed on the packaged breads, assessing the presence of *Salmonella* spp*.,* total and thermotolerant coliforms, mesophilic aerobes, molds, and yeasts. The results of these analyses are shown in Table 3.

**Table 3:** Microbiological Analysis of Breads^a^

films	Salmonella spp.	total coliforms (NMP/g)	thermotolerant coliforms (NMP/g)	mesophilic aerobes (CFU/g)	molds and yeasts (CFU/g)
F1	absence	ND	ND	2.5 × 10^4^ c	2.5 × 10^1^ b
F3	absence	ND	ND	25 × 10^5^ b	2.5 × 10^1^ b
F6	absence	ND	ND	1.25 × 10^6^ a	2.5 × 10^1^ b
F0	absence	ND	ND	2.5 × 10^2^ e	2.5 × 10^1^ b
C1	absence	ND	ND	2.5 × 10^3^ d	2.5 × 10^1^ b
C2	absence	ND	ND	2.5 × 10^6^ a	>2.50 × 10^6^ a
C3	NP	NP	NP	NP	NP

a NP: microbiological analyses were not performed because C3 was removed after 7 days of storage. ND: no contamination was detected. Values are expressed as means (*n* = 3). Means followed by the same letter on the same line are not statistically different from each other according to the Tukey test at a 5% significance level (*p* < 0.05). Formulations: F0 (without CC-0.5% CA), F1 (1% CC-0.25% CA), F3 (3% CC-0.5% CA), F6 (6% CC-1% CA), C1 (without CC and CA), C2 (low-density polyethylene), and C3 (unpackaged breads).

For *Salmonella* spp*.* research, the samples met the standards established by Brazilian legislation (RDC 724/2022).^26^ No presence of total or thermotolerant coliforms was detected in the samples. However, for mesophilic aerobes, the presence was detected with values ranging from 2.5 × 10^2^ to 2.5 × 10^6^ CFU/g. The total microbial load for formulations F6 and C2 was the highest among the samples for these microorganisms, classifying them as “unsatisfactory”, according to the criteria set by the National Institute of Health.^61^

Probably, the samples were contaminated by mesophilic aerobes, originating from the raw materials, handlers, or storage conditions. Therefore, formulations F6 and C2 were not effective in controlling mesophilic aerobic microorganisms. However, Brazilian legislation does not specify limits for mesophilic aerobic microorganisms in loaf-type bread. Similar results were observed by Al-Tayyar et al.^62^ when analyzing loaves of bread packaged in low-density polyethylene.

Formulation F6 exhibited a relatively high microbial count (1.25 × 10^6^ CFU/g); this result suggests that its antimicrobial action may have been limited by the high concentration of bioactive compounds, which could have compromised the integrity of the film polymeric matrix, reducing its barrier capacity against microorganisms. Jawad et al.,^35^ in their development of polymeric films containing acetyl-11-keto-β-boswellic acid (AKBA), boswellic acid, and silver nanoparticles, found that high concentrations of bioactive compounds caused significant structural alterations. The incorporation of these compounds led to reductions in tensile strength, moisture content, and water vapor permeability, all critical factors for maintaining the physical barrier’s effectiveness against microorganisms. These structural changes may have compromised the film’s ability to block contaminants, potentially limiting its antimicrobial effectiveness.

The water activity of films plays a crucial role in microbial growth, influencing both the functionality of the packaging and its antimicrobial efficacy. Formulations with higher concentrations of CC and CA exhibited increased water activity, which enhanced the antimicrobial interaction with bread. However, excessive water activity, driven by the hydrophilic compounds in the film, could compromise the film’s structure, ultimately affecting its performance as an antimicrobial packaging material.^35^ This indicates the need for formulation adjustments to optimize the antimicrobial effect while maintaining the material’s stability.

Additionally, substances with higher water activity may promote microbial growth, as bacteria generally require water activity values of at least 0.91 and fungi at least 0.6 to develop.^63^ Therefore, balancing the formulation of films is essential to ensure their functionality as active packaging, preventing the growth of undesirable microorganisms.

The films with natural additives were highly effective in delaying the development of molds and yeast, corroborating the results of the visual analysis discussed earlier (Figure 3). However, a count >2.5 × 10^6^ CFU/g of molds and yeast was observed in bread packed with the low-density polyethylene (C2) control film.

**Figure 4:**
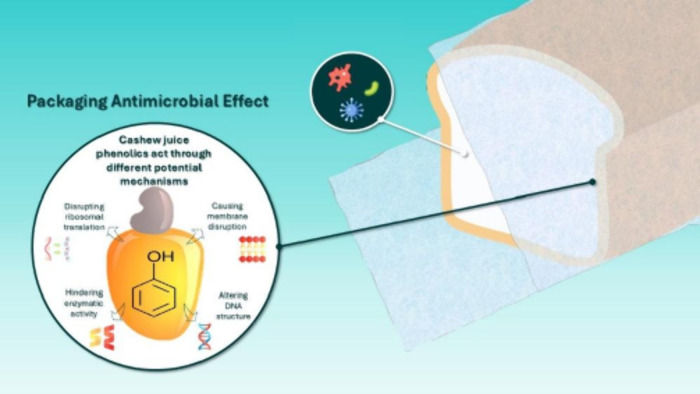
Schematics of possible antimicrobial mechanisms attributed to our developed active packaging.

Phenolic compounds exhibit antimicrobial activity through multiple mechanisms (Figure 4). These include the disruption of cell membranes, where they compromise membrane integrity, increase permeability, and induce cellular content leakage, ultimately leading to bacterial death. Phenolic compounds also affect DNA synthesis and regulation by binding to genomic DNA and generating hydrogen peroxide, causing oxidative damage. Furthermore, polyphenols inhibit bacterial metabolism by inactivating key enzymes through complexation with metal ions. Lastly, they modulate gene expression, downregulating virulence factors involved in toxin production, adhesion, motility, and invasion, thereby reducing bacterial pathogenicity.^11^ In addition, the carboxylic groups in citric acid can interact with microbial membranes, altering their permeability. This disruption can impede nutrient transport and lead to cell lysis, enhancing the antimicrobial effect.^13^

The migration of bioactive compounds from the packaging to the surface of the bread plays a crucial role in food preservation, as these compounds can directly interact with the substrate and inhibit microorganism growth.^64^ The incorporation of antimicrobial agents into active packaging has been extensively studied as an effective strategy to extend the shelf life of perishable products, allowing for the controlled release of active compounds into the environment or directly onto the food.^65^ Among the bioactive compounds used, cashew juice phenolic compounds stand out for their antimicrobial and antioxidant properties.^66^ These phenolic compounds possess the ability to interact with the food, promoting their gradual diffusion over time. This migration can be beneficial, as it allows for the continuous release of antimicrobial compounds, protecting against spoilage and pathogenic microorganisms, without the need for direct addition of preservatives to the food.^67^ However, it is essential to ensure that this migration occurs in a controlled manner, as excessive or improper release can compromise the stability of the packaging and affect the sensory properties of the food.^68^

Since bread is a relatively dry product, its low water activity may limit the antimicrobial action of the packaging, as many antimicrobial agents require sufficient moisture to activate and exert their antimicrobial properties.^69^ A promising approach to overcome this limitation is the development of controlled release systems that are responsive to environmental stimuli, a concept widely explored in areas such as biomaterials and pesticide release systems.^70^ These systems can be designed to release antimicrobial compounds selectively in response to factors such as relative humidity, temperature, light, pH, and enzymes.

However, the active packaging developed (F1 and F3) can be considered effective as antimicrobial packaging, as it successfully extended the shelf life of loaves of bread by up to four times compared to commercial packaging. The results indicated that microbial growth was inhibited by the films incorporated with natural additives. This effect is likely due to the release of phenolic compounds present on the surface of starch and pectin packages, which can denature membrane proteins of microorganisms through hydrogen bonds, alter membrane permeability, and inhibit nucleic acid production, ultimately leading to cell death.^11^

## 4 Conclusions

The objective of developing an antimicrobial active packaging for application in bread was successfully achieved. Formulations containing the lowest concentrations of cashew bioactive compounds (CC) and citric acid (CA), specifically F1 and F3, promoted the production of films with improved physical, barrier, chemical, and thermal characteristics. The inclusion of these additives increased film flexibility but led to reduced tensile strength and increased water vapor permeability compared to the control films. Despite the rise in permeability, the antimicrobial effectiveness of F1 and F3 packaging was not compromised.

The formulated films exhibited promising attributes, including a homogeneous appearance and low water solubility, which support their application for specific food preservation needs. When used to package bread, these films were effective in maintaining microbial stability extending the product’s shelf life from 7 days to 28 days.

Thus, the antimicrobial active packaging developed in this study aligns with consumer preferences for safer, additive-free foods by delivering natural preservatives through innovative packaging. Furthermore, these active films present a sustainable alternative to conventional passive packaging and offer a means of valorizing cashew byproducts, contributing to reduced environmental impact associated with petroleum-based materials.

## Data Availability

Availability of data and materials data are available by contacting the authors.
